# Metabolism of 20(*S*)-Ginsenoside Rg_2_ by Rat Liver Microsomes: Bioactivation to SIRT1-Activating Metabolites

**DOI:** 10.3390/molecules21060757

**Published:** 2016-06-10

**Authors:** Li-Yuan Ma, Qi-Le Zhou, Xin-Bao Yang, Hong-Ping Wang, Xiu-Wei Yang

**Affiliations:** State Key Laboratory of Natural and Biomimetic Drugs, Department of Natural Medicines, School of Pharmaceutical Sciences, Peking University Health Science Center, Peking University, No. 38, Xueyuan Road, Haidian District, Beijing 100191, China; maliyuan0506@126.com (L.-Y.M.); zqlzdn@163.com (Q.-L.Z.); xbyang0718@163.com (X.-B.Y.); sungirl9626@163.com (H.-P.W.)

**Keywords:** 20(*S*)-ginsenoside Rg_2_, ginsenotransmetin A, liver microsomes, SIRT1 activator

## Abstract

20(*S*)-Ginsenoside Rg_2_ (**1**) has recently become a hot research topic due to its potent bioactivities and abundance in natural sources such as the roots, rhizomes and stems-leaves of *Panax* *ginseng*. However, due to the lack of studies on systematic metabolic profiles, the prospects for new drug development of **1** are still difficult to predict, which has become a huge obstacle for its safe clinical use. To solve this problem, investigation of the metabolic profiles of **1** in rat liver microsomes was first carried out. To identify metabolites, a strategy of combined analyses based on prepared metabolites by column chromatography and ultra-performance liquid chromatography coupled with quadrupole time-of-flight tandem mass spectrometry (UPLC-Q-TOF/MS) was performed. As a result, four metabolites **M1**–**M4**, including a rare new compound named ginsenotransmetin A (**M1**), were isolated and the structures were confirmed by spectroscopic analyses. A series of metabolites of **1**, **M_A_**–**M_G_**, were also tentatively identified by UPLC-Q-TOF/MS in rat liver microsomal incubate of **1**. Partial metabolic pathways were proposed. Among them, **1** and its metabolites **M1**, **M3** and **M4** were discovered for the first time to be activators of SIRT1. The SIRT1 activating effects of the metabolite **M1** was comparable to those of **1**, while the most interesting SIRT1 activatory effects of **M3** and **M4** were higher than that of **1** and comparable with that of resveratrol, a positive SIRT1 activator. These results indicate that microsome-dependent metabolism may represent a bioactivation pathway for **1**. This study is the first to report the metabolic profiles of **1** *in* *vitro*, and the results provide an experimental foundation to better understand the *in vivo* metabolic fate of **1**.

## 1. Introduction

**Scheme 1:**
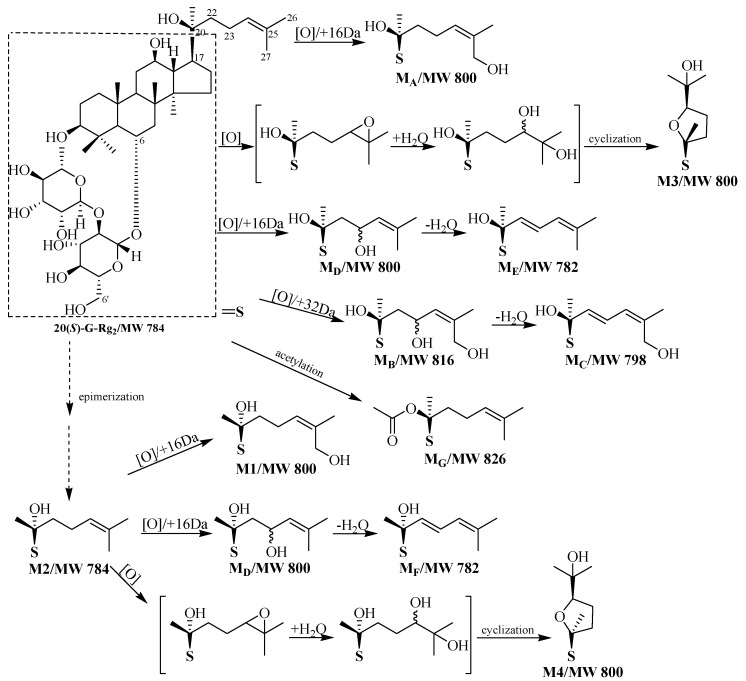
Chemical structures of 20(*S*)-G-Rg_2_, **M1**–**M4**, **M_A_**–**M_G_** and proposed possible metabolic pathways.

Currently worldwide public demand for ginseng, the roots and rhizomes of *Panax ginseng* C. A. Meyer (family Araliaceae), is rapidly increasing. Ginseng is sold as a food additive in the U.S. and, thus, it need not meet the specific safety and efficacy requirements required of drugs by the Food and Drug Administration [1]. There are active chemical ingredients called ginseng saponins (ginsenosides) in ginseng, which has been reported to be responsible for ginseng’s biological activities which include immune enhancement, antioxidant and memory enhancement effects, the recovery of vital energy, the alleviation of fatigue, blood flow improvement, and promoting longevity [2,3,4]. 20(*S*)-ginsenoside Rg_2_ (20(*S*)-G-Rg_2_; **1**, Scheme 1) is a well-known bioactive saponin of the roots and rhizomes [5], and the stems/leaves [6,7] of ginseng as well as red ginseng [8], and it is abundantly available from natural sources like the stems/leaves of *P. ginseng*. Modern pharmacological studies have demonstrated that **1** exhibits excellent activity against cardio-cerebrovascular diseases [3,4,9,10,11,12]. Absorption, distribution, metabolism, excretion and toxicity (ADMET) studies have been introduced into the earlier stages of drug discovery instead of a serial strategy, because ADMET and pharmacokinetic issues are often responsible for new drug failures in clinical trials. It is therefore important to investigate the metabolic characteristics of **1**. It has been reported that **1** can be absorbed into the systemic circulation after oral administration of total saponins from the stems/leaves of *P. ginseng* [13] and eliminated after intravenous administration of individual 20(*R*)- and 20(*S*)-ginsenosides Rg_2_ [14,15] to rats. Up to now, however, its metabolic fate remains unknown. Therefore, a study of the metabolism of **1** is indispensable.

The main purpose of this study was to investigate the *in vitro* metabolic fate of **1** treated with rat liver microsomes to understand the molecular structural diversity of the metabolites. Owing to their low concentration, identification of some metabolites presents a great challenge. Ultra-high performance liquid chromatography coupled with quadrupole time-of-flight tandem mass spectrometry (UPLC-Q-TOF/MS) [16,17,18] has become a routine tool for the detection and identification of predictable and unpredictable metabolites, because of its high-resolution and the fact it can provide molecular formulas as well as exact molecular masses. In order to identify the predictable and unpredictable metabolites, extraction ion chromatographies (EIC) based on high-resolution LC-MS data was employed in this study. We successfully identified a prototype compound and four metabolites, including one new compound, by extensive spectroscopic data analyses as well as seven other ones by UPLC-Q-TOF/MS data analyses in rat liver microsomal incubate.

The role of metabolic reactions, resulting in bioactive metabolites of **1**, is not well defined as yet. The metabolism of **1** in the liver could reflect a bioactivation pathway resulting in the formation of bioactive metabolites. On the other hand, silent information regulator two homolog 1 (SIRT1) is a member of the sirtuin family that possesses NAD^+^-dependent deacetylase activity [19] and regulates a variety of cellular processes such as energy metabolism, cell-cycle progression [20], tumors [20,21,22] and aging [23]. In addition, it has been reported that modulation of SIRT1 may offer novel therapeutic options for targeting cardiovascular risk factors [24]. SIRT1 is becoming an important target for new therapies in the treatment of some diseases. Ginseng has been used for a panacea or promoting longevity. To further explore the anti-aging potential of ginseng [25,26], the SIRT1 promotion activity of **1** was evaluated using a SIRT1 fluorescence activity assay kit. In this assay, resveratrol was used as a positive activator [27,28,29] and nicotinamide was used as a positive inhibitor [30].

## 2. Results and Discussion

Even though the LC-MS technique has become a routine tool for the identification of the metabolites in bio-samples [16,17,18], a more precise approach to obtain reliable chemical structure information of a pure metabolite is NMR experiments, specifically several multidimensional NMR spectra are combined for resonance assignment. Therefore, the strategy for characterizing the metabolites of **1** in rat liver microsomal incubate was divided into three steps, including (1) the metabolites were isolated and purified by open column chromatographic (CC) and reversed-phase semipreparative high performance liquid chromatography (RP-SP-HPLC) methods; (2) the chemical structures of the metabolites were identified by extensive spectroscopic data analyses and comparing with the reference standards as well as matching their retention time (t_R_); (3) tentatively characterization of the trace metabolites based on exact molecular weights (four decimal places) and MS^n^ fragmentions.

**Figure 1:**
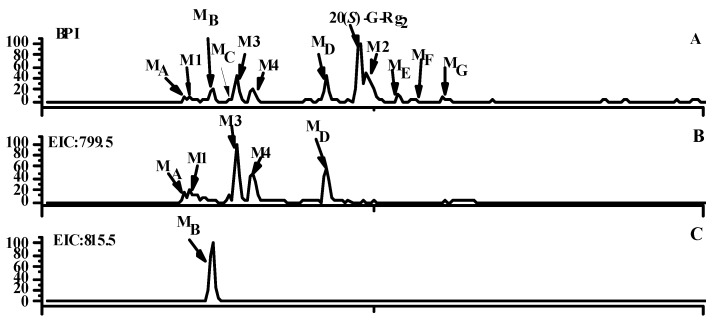

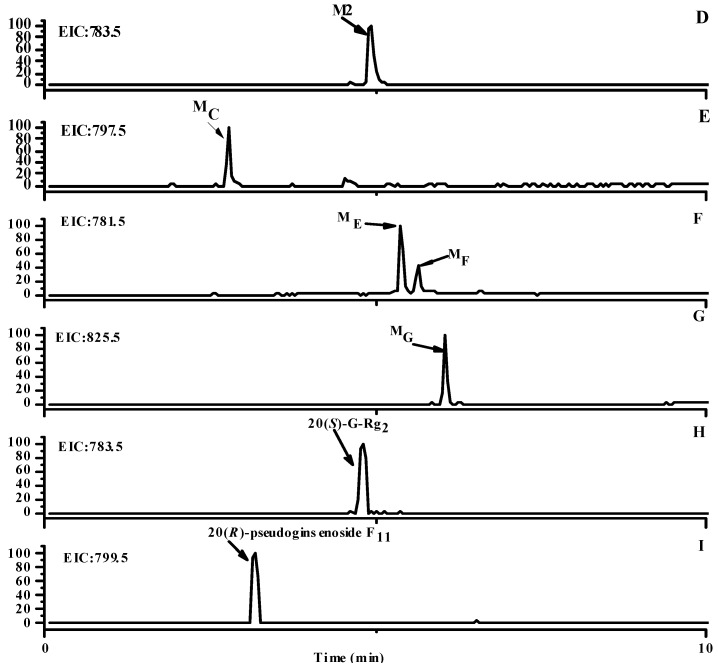
The typical base peak intensity (BPI) chromatograms of 20(*S*)-G-Rg_2_ metabolites. (**A**) BPI of all metabolites; (**B**) EIC of **M1**, **M3**, **M4**, **M_A_** and **M_D_**; (**C**) EIC of **M_B_**; (**D**) EIC of **M2**; (**E**) EIC of **M_C_**; (**F**) EIC of **M_E_** and **M_F_**; (**G**) EIC of **M_G_**; (**H**) EIC of authentic 20(*S*)-G-Rg_2_; (**I**) EIC of authentic 20(*R*)-pseudoginsenoside F_11_.

In the optimization of ESI-MS for the analysis of metabolites, the four reference standard ginsenosides, 20(*S*)-G-Rg_2_, 20(*R*)-G-Rg_2_, pseudoginsenoside F_11_ and 20(*R*)-pseudoginsenoside F_11_, were detected in both positive and negative ion modes. Compared to the positive mode, the MS spectrum of each ginsenoside showed lower baseline noise and better ionization effect in the negative ion mode, which made it superior for identifying the metabolites of 20(*S*)-G-Rg_2_. According to the t_R_, ESI-MS (molecular weight) and MS/MS (fragment ion) information, the chromatographic behaviors and the MS spectra of the four reference standards were characterized, which were the basis for identifying the metabolites in rat liver microsomal incubate. The negative MS/MS spectra were obtained from the deprotonated molecular ion [M − H]^−^, and the mass spectra of product ion of [M − H]^−^ exhibited a fragmentation pattern corresponding to the successive loss of the glycosidic (rhamnopyranosyl, rha; glucopyranosyl, glc) units till the formation of *m/z* 391 fragmention ion [M − partial C_17_-side-chain − H]^−^. The typical mass spectra and possible fragmentations of 20(*S*)-G-Rg_2_ (A) and pseudoginsenoside F_11_ (B) are shown in Figure S1. As a result, a total of 11 metabolites were confirmed and their EIC are shown in Figure 1A–I.

### 2.1. Identification of Metabolites ***M1**–**M3***

The metabolism of 20(*S*)-G-Rg_2_ (**1**) was studied by its *in vitro* incubation with liver microsomes from sodium phenobarbital (PB) pre-treated male rats. Under the optimized system [31], butanol (BuOH) extract of the metabolites of **1** was subjected to open silica gel CC and RP-SP-HPLC to yield three metabolites **M1**–**M3** and the parent compound **1**. The structures of the metabolites were determined to be as shown in Scheme 1 by spectroscopic methods. The chemical structures of prototype **1** [7], **M2** and **M3** were determined by extensive NMR and MS data analyses as well as comparison with the reference standards. **M2** and **M3** were unambiguously identified as 20(*R*)-G-Rg_2_ [7] and pseudoginsenoside F_11_ [32]. For the NMR data of **1** and **M3** see Supplementary information Table S1.

**M1** was isolated as a white amorphous powder with an ${\left[\alpha \right]}_{D}^{20}$ +12.8 (*c* 0.185, MeOH). Its molecular formula was assigned to be C_42_H_72_O_14_ based on a quasi-molecular ion peak [M + HCOOH − H]^−^ at *m/z* 845.4896 in the negative HR-ESI-MS and NMR spectroscopic data, which was one oxygen atom more than the prototype **1**. Its IR spectrum showed maximum absorption bands for hydroxyl and ether bond functions at 3417 and 1075 cm^−1^, respectively. Complete unambiguous assignments for the ^1^H- and ^13^C-NMR signals (Table 1 and Table 2) of **M1** were made by combination of ^1^H–^1^H COSY, HSQC, HMBC, and NOESY spectra.

**Figure 2:**
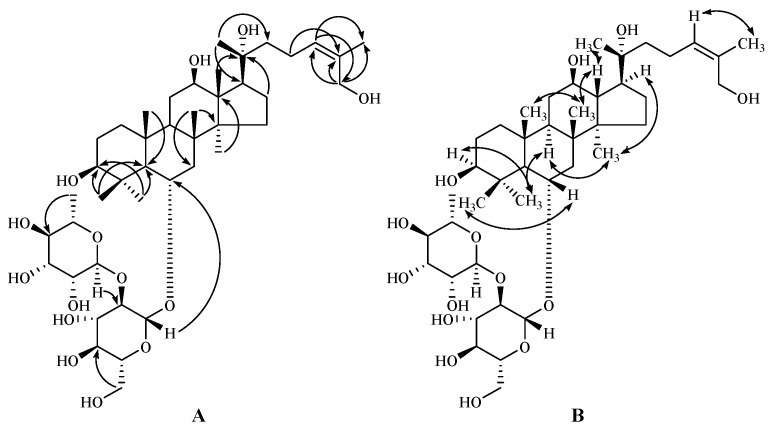
Key HMBC (from H to C, **A**) and NOESY (**B**) correlations of **M1**.

As found in **1** and **M2**, the ^1^H- and ^13^C-NMR spectra of **M1** indicated a dammarane-type triterpene with a glucopyranosyl and a rhamnopyranosyl moieties [7], and showed similarity except for the signals for a hydroxymethyl group instead of a methyl group at C-17 side-chain. The Me-26 proton signal at δ_H_ 1.63 (3H, s) in the **M2** was shifted downfield to δ_H_ 1.96 (3H, s) in the **M1**, and H-24 at δ_H_ 5.24 (1H, t, *J* = 6.8 Hz) in the **M2** was shifted downfield to δ_H_ 5.43 (1H, t, *J* = 7.5 Hz) in the **M1**. In the ^13^C-NMR spectrum, the signal at *δ*_C_ 17.8 for C-27 in **M2**, was replaced by a signal at δ_C_ 61.0 in **M1**, suggesting that the Me-27 of **M2** was substituted by a hydroxymethyl group. The molecular formula, C_42_H_7__2_O_14_, of **M1** contains one more oxygen than those of **1** and **M2**, which further confirmed that **M1** was a hydroxymethyl group-substituted product of **1** or **M2**. This conclusion was firmly supported by the correlations between H-24 at δ_H_ 5.43 and C-26 at δ_C_ 22.0, C-27 at δ_C_ 61.0 in the HMBC experiment (Figure 2A). The stereochemistry at C-20 was assigned as *R*, due to signals at δ_C_ 50.7 for C-17, δ_C_ 22.0 for C-21 and δ_C_ 42.9 for C-22 [7]. This conclusion was also supported by a cross peak between δ_H_ 1.29 (Me-21) and δ_H_ 1.92 (H-13β) in a 2D NOESY experiment of **M1** (Figure 2B).

In addition, the signals of Me-26 proton at δ_H_ 1.96 and H-24 at δ_H_ 5.43 showed an NOE correlation, whereas the signals of the H-27 at δ_H_ 4.44, 4.51 and H-24 at δ_H_ 5.43 showed no NOE correlation in the 2D NOESY spectrum of **M1**, indicating that the double bond in **M1** has a *Z*-configuration. Considering of all the above data, **M1** was elucidated as 6-*O*-α-l-rhamnopyranosyl-(1→2)-β-d-glucopyranosyl- dammar-24*Z*-ene-3β,6α,12β,20(*R*),27-pentol and given the trivial name ginsenotransmetin A. As far as we know, this is the first report on the identification of **M1**.

Aside from the above-mentioned three metabolites **M1**–**M3**, the additional metabolite **M4** in the rat liver microsomal incubate of **1** was identified as 20(*R*)-pseudoginsenoside F_11_ [32] by matching *t*_R_, the empirical molecular formula and diagnostic fragment ions with those of an authentic sample.

### 2.2. Identification of Metabolites ***M_A_**–**M_G_***

The BuOH extract of the microsomal incubate of **1** was dissolved in MeOH and then injected into the UPLC–Q-TOF/MS system. The typical base peak intensity (BPI) are shown in Figure 1A. A total of 11 metabolites profile were displayed.

**Figure 3:**
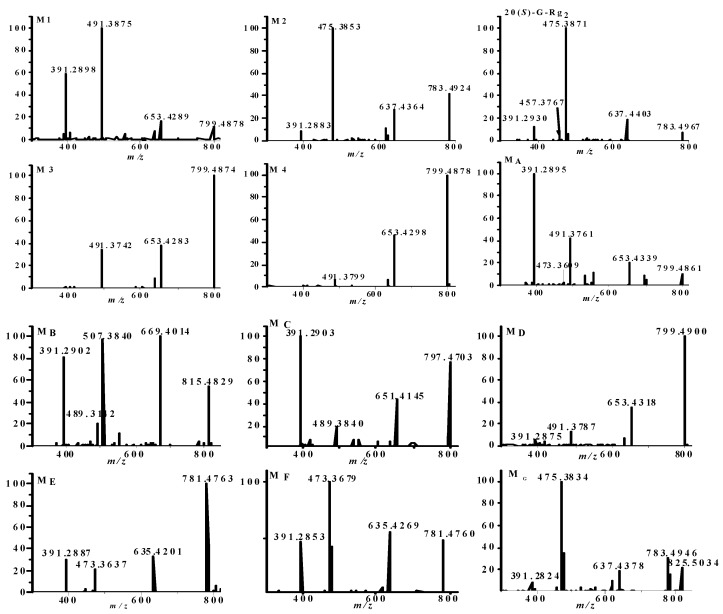
The MS^2^ spectra of 20(*S*)-G-Rg_2_, **M1**–**M4** and **M_A_**–**M_G_**.

**M_A_** was detected at 2.15 min. Its molecular formula was assigned to be C_42_H_72_O_14_ based on a quasi-molecular ion peak [M − H]^−^ at *m/z* 799.4861 in HR-ESI-MS, which indicates one oxygen atom more than prototype compound **1** and the same molecular formula as **M1**. The MS^2^ spectrum (Figure 3) showed fragment ions at *m/z* 799.4861 (C_42_H_7__1_O_14_) [M − H]^−^, 653.4339 (C_36_H_6__1_O_10_) [M − rha − H]^−^, 491.3761 (C_30_H_5__1_O_5_) [M − glc − rha − H]^−^, 473.3609 (C_30_H_49_O_4_) [M − glc − rha − H_2_O − H]^−^, and 391.2895 (C_24_H_39_O_4_) [M − glc − rha − H_2_O − C_6_H_10_ − H]^−^, suggesting that the mono-oxygenation reaction had occurred at the end of the C_17_-side-chain, a reaction that may be catalyzed by CYP450-dependent terminal hydroxylases in the liver [33]. It has been reported that 20(*S*)-type ginsenosides always elute before 20(*R*)-type ginsenosides during analysis by reversed-phase HPLC on a C_18_ column [34]. Thus, **M_A_** was tentatively identified as a 20(*S*)-epimer of **M1**, namely quinquenoside L_11_ [35].

**M_B_** was detected at 2.58 min. Its molecular formula was assigned to be C_42_H_72_O_15_ based on a quasi-molecular ion peak [M − H]^−^ at *m/z* 815.4829 in HR–ESI-MS, which has two oxygen atoms more than the prototype compound **1** and one oxygen atom more than **M_A_**. The MS^2^ spectrum (Figure 3) showed fragment ions at *m/z* 815.4829 (C_42_H_7__1_O_15_) [M − H]^−^, 669.4014 (C_36_H_61_O_11_) [M − rha − H]^−^, 507.3840 (C_30_H_51_O_6_) [M – glc − rha − H]^−^, 489.3142 (C_30_H_49_O_5_) [M − glc − rha − H_2_O − H]^−^, and 391.2902 [M − glc − rha − H_2_O − C_6_H_10_O − H]^−^, suggesting that the dioxygenation reaction had occurred on the C_17_-side-chain. It has been discovered that hydroxylation reaction of C-23 was very easier than that of C-22 [36], therefore, **M_B_** was tentatively identified as 23-hydroxyquinquenoside L_11_.

**M_C_** was detected at 2.79 min, with a molecular formula of C_42_H_70_O_14_ determined by HR-ESI-MS from a quasi-molecular ion peak [M − H]^−^ at *m/z* 797.4703, which was 14Da more than prototype compound **1**. The MS^2^ spectrum (Figure 3) showed fragment ions at *m/z* 797.4703 (C_42_H_69_O_14_) [M − H]^−^, 651.4145 (C_36_H_59_O_10_) [M − rha − H]^−^, 489.3840 (C_30_H_49_O_5_) [M − glc − rha − H]^−^, 391.2903 (C_24_H_39_O_4_) [M − glc − rha − C_6_H_10_O − H]^−^, suggesting that the monooxygenation reaction had occurred at the end of the C_17_-side-chain as found in **M_A_** and dehydration at C-22, C-23. Consequently, the structure of **M_C_** was tentatively concluded to be 6-*O*-α-l-rhamnopyranosyl-(1→2)-β-d-glucopyranosyl-dammar- 22,24-diene-3β,6α,12β,20(*S*),27-pentol.

**M_D_** was detected at 4.29 min and had the molecular formula C_42_H_72_O_14_ by HR-ESI-MS from a quasi-molecular ion peak [M − H]^−^ at *m/z* 799.4900, which was one oxygen atom more than prototype **1**. The MS^2^ spectrum (Figure 3) showed fragment ions at *m/z* 799.4900 (C_42_H_7__1_O_14_) [M − H]^−^, 653.4318 (C_36_H_61_O_10_) [M − rha − H]^−^, 491.3787 (C_30_H_51_O_5_) [M − glc − rha − H]^−^, 391.2875 (C_24_H_39_O_4_) [M − glc − rha − C_6_H_12_O − H]^−^, suggesting that the monooxygenation reaction had occurred at the C_17_-side-chain and a hydroxyl group could be added at C-23 [36]. This conclusion was also supported by the identification of **M_E_**. Finally, **M_D_** was tentatively identified as 6-*O*-α-l-rhamnopyranosyl-(1→2)-β-d-glucopyranosyl-dammar-24-ene-3β,6α,12β,20(*S* or *R*),23-pentol.

**M_E_** was detected at 5.36 min. The molecular formula of **M_E_** was deduced as C_42_H_70_O_13_ from its a quasi-molecular ion peak [M − H]^−^ at *m/z* 781.4763 in HR-ESI-MS. The mass was found to be 2Da less than prototype **1**. The MS^2^ spectrum (Figure 3) showed fragment ions at *m/z* 781.4763 (C_42_H_69_O_13_) [M − H]^−^, 635.4201 (C_36_H_59_O_9_) [M − rha − H]^−^, 473.3637 (C_30_H_49_O_4_) [M − glc − rha − H]^−^, 391.2887 (C_24_H_39_O_4_) [M − glc − rha − C_6_H_10_ − H]^−^, suggesting **M_E_** was a dehydrogenated derivatives of prototype **1** in the C_17_-side-chain. Accordingly, the structure of **M_E_** was established as 22,23-dehydro-20(*S*)-ginsenoside Rg_2_. It is a dehydration product of **M_D_**.

**M_F_** was eluted at 5.65 min. The molecular formula of M**_F_** was established as C_42_H_70_O_13_ by HR-ESI-MS showing a quasi-molecular ion [M − H]^−^ peak at *m/z* 781.4760, which is the same molecular formula as **M_E_**. The MS^2^ spectrum (Figure 3) showed fragment ions at *m/z* 781.4760 (C_42_H_69_O_13_) [M − H]^−^, 635.4269 (C_36_H_59_O_9_) [M − rha − H]^−^, 473.3679 (C_30_H_49_O_4_) [M − glc − rha − H]^−^, 391.2853 (C_24_H_39_O_4_) [M − glc − rha − C_6_H_10_ − H]^−^, and had the same fragment ions as **M_E_**. Because the *t*_R_ of **M_F_** was longer than that of **M_E_** as in **M_A_** behavior, the structure of **M_F_** was concluded to be a 20(*R*)-epimer of **M_E_**, namely 22,23-dehydro-20(*R*)-ginsenoside Rg_2_.

**M_G_** was eluted at 6.04 min with a deprotonated molecular ion [M − H]^−^ peak at *m/z* 825.5034, corresponding to a molecular formula of C_44_H_74_O_14_. The MS^2^ spectrum (Figure 3) showed fragment ions at *m/z* 825.5034 (C_44_H_7__3_O_14_) [M − H]^−^, 783.4946 (C_42_H_71_O_13_) [M − Ac − H]^−^, 637.4378 (C_36_H_61_O_9_) [M − Ac − rha − H]^−^, 475.3834 (C_30_H_51_O_4_) [M − Ac − glc − rha − H]^−^, 391.2824 (C_24_H_39_O_4_) [M − Ac − glc − rha − C_6_H_12_ − H]^−^. Because its mass was found to be 42Da higher than prototype **1**, the structure of **M_G_** was concluded to be an acetylated derivatives of prototype **1**, but the location of the acetyl group remained ambiguous (C-20 of aglycone or C-6′ of glucosyl group).

### 2.3. Analysis of Metabolic Pathways

The prototype and related metabolites identified in this research provide a global view of metabolite profiles of **1** in rat liver microsomes. From the results of metabolite characterization, we discovered that majority of **1** metabolites correspond to oxidative metabolites and they are phase I liver metabolites. The partial metabolic pathways shown in Scheme 1 are proposed according to the chemical diversity of the metabolites and the properties of drug metabolism in liver microsomes [37,38,39,40].

A total of seven oxidative metabolites (**M1**, **M3**, **M4**, and **M_A_**–**M_D_**) were detected and characterized by their MS fragmentation patterns using LC-ESI MS^n^. Among them, the chemical structures of **M1** and **M3** were unambiguously determined by extensive 1D and 2D NMR data analyses, and **M4** was unambiguously determined by comparison with an authentic sample. The specific forms of CYP450 involved in different monooxygenation reactions have been previously characterized [37,38,39,40], especially, cleavage of the C_17_-side chain may be catalyzed by a specific CYP450 enzyme, CYP11A1 [37]. Interestingly, all oxidative modifications occurred at the C_17_-side-chain of the dammarane aglycone moiety and all metabolites had the same intact sugar chain. Because the relative yield of **M2** was higher in all metabolites, it might seem that epimerization of C_20_-OH was the initial steps in **1** metabolism to produce **M2**, followed by a monooxygenation reaction to generate metabolite **M1**, so we propose that the metabolism pathway was that 20(*S*)-ginsenoside Rg_2_ was firstly converted to any intermediates and further converted to 20(*R*)-ginsenoside Rg_2_. The details needs further study. Simultaneously, a monooxygenation reaction also occurred at the vinyl methyl group in the C_17_-side-chain of prototype **1** to generate **M_A_**, indicating that hydroxylation of the vinyl methyl group in the C_17_-side-chain was easier. A monooxygenation reaction of **1** involving C-23 produced the metabolite **M_D_**, followed through a dehydration to generate a metabolite **M_E_**. **M2** was consecutively mono-oxygenated and dehydrated to form **M_F_** similar to the steps of **M_E_**.

The liver microsomal enzymes can also produce dioxygenation reactions [41]. A dioxygenation reaction of **1** involving C-23 and C-27, respectively, generates the metabolite **M_B_**, followed by a dehydration reaction to produce another metabolite, **M_C_**.

The chemical structure of **M3** was unambiguously determined as pseudoginsenoside F_11_ [32] by extensive 1D and 2D NMR data analyses (see Supplementary Materials Table S1) and a comparison with a reference standard. It was a well-known ocotillone-type ginsenoside. The C_17_-side-chain double bond of prototype **1** was epoxidized to produce a 24,25-epoxide derivative followed by a subsequent catalytic hydrolysis to form a 24,25-dihydroxyl compound (undetectable because of the quick rearrangement) and cyclization to form **M3**. **M2** possibly undergoes a similar metabolism to generate **M4**.

From all the results above, it could be found that the abundance of identified mono-oxygenated metabolites reflected the quantity of dioxygenated metabolites and 27-hydroxylation occurred to the greatest extent and hydroxylation occurred preferably at C-25 than at C-23 and in addition a small amount of metabolites **M_C_**, **M_E_** and **M_F_** with a conjugated double bond system were formed.

### 2.4. Bioactivation of 20(S)-G-Rg_2_ to Its Metabolites by Microsomal Metabolism

**Figure 4:**
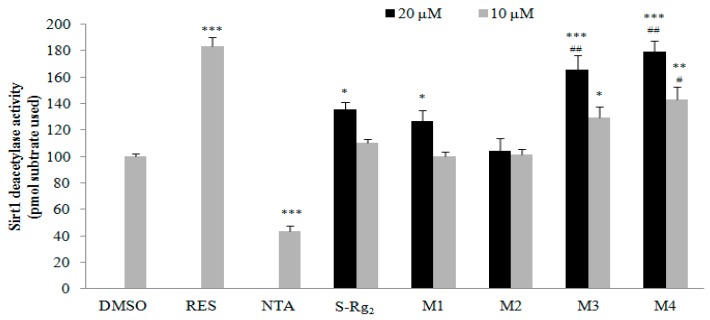
Effects of 20(*S*)-G-Rg_2_ (S-Rg_2_) and its metabolites **M1**, **M2**, **M3**, **M4** on SIRT1 activity. RES (resveratrol, 10 μM) was used as a positive activator and NTA (nicotiamide, 10 μM) was used as a positive inhibitor. For all assays, the tested compounds were dissolved in dimethyl sulfoxide (DMSO) and the final DMSO concentration was 0.1% (*v*/*v*). * *p* < 0.05, ** *p* < 0.01, *** *p* < 0.001 compared with DMSO (control) group in three separate experiments. ^#^ *p* < 0.05, ^##^ *p* < 0.01 compared with S-Rg_2_ group in three separate experiments.

Ginseng has been used for more than 3000 years in the belief that it is a panacea and promotes longevity. How to control aging and longevity has long been a topic of considerable interest. SIRT1 activator is believed to extend lifespan [42,43]. It has been reported that ginsenosides Rb_1_ [44], Rc [45], Rh_3_ [46], 20(*S*)-ginsenoside Rg_3_ [47,48], 6α,20(*S*)-dihydroxydammar-3,12-dione-24-ene, 6α,20(*S*),24(*S*)-trihydroxydammar-3,12-dione-25-ene, 6α,20(*S*),25-trihydroxydammar-3,12-dione-23-ene, dammar-20(22),24-diene-3β,6α,12β-triol [48], and red ginseng extract [49] showed potential as SIRT1 activators. However, it is unknown whether **1** is involved as a SIRT1 activator. To investigate the bioactivation of **1** to its metabolites by microsomal metabolism, the SIRT1 promotion activity of **1** and its selected metabolites was evaluated using a SIRT1 fluorescence activity assay kit [47,50,51,52,53], which is a well-known model for anti-aging evaluation. The results are shown in Figure 4. Based on the protocols provided with the kit instructions, the assay compounds were used at concentrations of 10 and 20 µM. The prototype **1** and its metabolites **M1**, **M3** and **M4** could stimulate SIRT1 activity in a concentration-dependent manner at 10 and 20 µM, indicating they are potential SIRT1 activators. The metabolite **M1** stimulated SIRT1 activity at a concentration of 20 µM comparably to **1**. The metabolites **M3** and **M4** showed the most potent stimulatory effect, higher than that of **1**, and comparable with that of resveratrol used as a positive control SIRT1 activator [27,28,29]. These results indicated that liver microsome enzyme-dependent metabolism may represent a bioactivation pathway that alters the bioactivity of **1**. The metabolic conversion may be essential for the therapeutic actions of **1**, and the effect of **1** for extending lifespan may be due to its activation of the SIRT1 pathway. 

Whether these superior benefits will actually translate to humans requires further study. Because of the limited amounts of the individual compounds available from this investigation, the metabolites M_A_–M_D_ could not be assayed for SIRT1 activity.

## 3. Experimental Section

### 3.1. Materials

β-Nicotinamide adenine dinucleotide phosphate (NADP), reduced nicotinamide adenine dinucleotide (NADH), glucose-6-phosphate (G-6-P) and glucose-6-phosphate dehydrogenase (G-6-PDH) were purchased from Sigma Chemical Co. (St. Louis, MO, USA). Sodium phenobarbital (PB) was purchased from Peking University Third Hospital (Beijing, China). LC-MS grade acetonitrile (MeCN) and methanol (MeOH) were purchased from J. T. Baker (Phillipsburg, NJ, USA), and LC-MS grade formic acid was obtained from Fisher-Scientific (Fair Lawn, NJ, USA). Ultrapure water (H_2_O) was prepared by a Milli-Q system (Millipore, Billerica, MA, USA) in our laboratory. Other reagents were of analytical grade.

20(*S*)-G-Rg_2_ (**1**) and 20(*R*)-G-Rg_2_ were isolated and purified from *P. ginseng* as described in previous papers [6,7]. Reference standard of pseudoginsenoside F_11_ was purchased from Chengdu Must Bio-Technology Co., Ltd. (Chengdu, China) and 20(*R*)-pseudoginsenoside F_11_ which was isolated and purified from red American ginseng and published in a reference [31] was a gift from Professor Ping-Ya Li of the Institute of Frontier Medical Science, Jilin University (Changchun, China).

Optical rotations were measured on an Autopol III polarimeter (Rudolph Research Analytical, Flanders, NJ, USA) using MeOH as solvent. Infrared (IR) spectrum was recorded on a Nexus-470 FT-IR spectrometer (Thermo Nicolet, Inc., Madison, WI, USA) with KBr disks. Mass spectra were recorded on an API QSTAR (Applied Biosystems/MDS Sciex., Foster City, CA, USA) for ESI-TOF-MS and a Waters Xevo G2 Q-TOF mass spectrometer (Waters, Milford, MA, USA) for HR-ESI-MS. One- and two-dimensional (1D and 2D) NMR spectra were performed on a Bruker AVANCE III 400 spectrometer (400 MHz for ^1^H and 100 MHz for ^13^C; Bruker BioSpin AG Facilities, Fällanden, Switzerland) with tetramethylsilane as an internal standard and pyridine-*d*_5_ (py-*d*_5_) as the solvent. RP-SP-HPLC was conducted on a Beijing CXTH 3000 system (Beijing Chuang Xin Tong Heng Sci. Technol. Co. Ltd., Beijing, China) with two P3050 pumps, UV3000 ultraviolet-visible detector, A1359 liquid handler, Daisogel C_18_ column (205 mm × 30 mm, 10 µm), and all UV detection was set at 203 nm with a flow rate of 15 mL/min. CC separation was carried out using silica gel (200–300 mesh; Qingdao Marine Chemical Co., Qingdao, China) as the stationary phase, and thin layer chromatography (TLC) was conducted on silica gel GF_254_ plates (Merck, Darmstadt, Germany).

### 3.2. Preparation of Rat Liver Microsomes

Male Sprague–Dawley rats weighting 200–220 g were supplied by the Laboratory Animal Center of Peking University Health Science Center (Beijing, China). The rats were kept in a controlled breeding room with temperature conditions at 22 ± 1 °C and relative humidity at 60% ± 5% before the start of the experiment. They were fed standard laboratory chow with water *ad libitum* for 3 days and fasted overnight prior to the experiment. All experimental procedures were approved by the Animal Care Ethics committee on Peking University (No. LA2014162) and conducted according to the European Community guidelines for the use of experimental animals. To induce cytochrome P450 enzymes (CYP450), rats were given PB in physiological saline intraperitoneally as a single dose of 60 mg/kg body weight and sacrificed on day 3 after treatment. Their livers were quickly removed and then washed with ice-cold 10 mM potassium phosphate buffer (PPS; pH 7.4), and kept ice-cold. The livers were sliced and thoroughly homogenized with four-volumes of PPS (pH 7.4) using an F6/10 superfine homogenizer (Fluko equipment, Shanghai, China), and centrifuged at 12,500 *g* for 15 min at 4 °C using a GL-20B centrifuge (Anke equipment, Shanghai, China) to produce a supernatant. The supernatant was transferred to ultracentrifuge tubes and then centrifuged at 105,000 *g* for 65 min at 4 °C using an LB-80M ultracentrifuge (Beckman Instrument Inc., Fullerton, CA, USA). The microsomal pellets were resuspended in 50 mM PPS (pH 7.4). The amount of microsomal protein was determined following the method of Lowry-Folin [54]. CYP450 concentrations were determined as described by a reference [55]. Rat liver microsomes (RLM) were found to contain 1.09 nM (18.55 mg proteins/mL) CYP450 per mg protein.

### 3.3. 20(S)-G-Rg_2_ Metabolism by Rat Liver Microsomes

Under the optimized system [31], complete incubation systems (final volume, 2000 mL) contained RLM (up to 2.0 mg/mL), 100 mM phosphate buffer (pH 7.4), 20(*S*)-G-Rg_2_ of 0.15 mg/mL, and an NADPH-generating system consisting of 10.0 mM G-6-P, 1.0 mM NADP, 0.5 mM NADH, G-6-PDH of 1.0 IU/mL and 4.0 mM MgCl_2_. After RLM and 20(*S*)-G-Rg_2_ were preincubated for 5 min, the reaction was started by the addition of the NADPH-generating system. Incubations were performed at 37 °C for 120 min with continuous shaking (100 rpm) in a Dubnoff incubator. Reaction was terminated by adding ice-cold cyclohexane (500 mL). After removal of the cyclohexane layer, the aqueous layer was extracted six times with an equal volume of BuOH. The BuOH extracts were combined and then concentrated by evaporation under vacuum to give 22.85 g of residue.

### 3.4. Isolation and Purification of Metabolites

The above-mentioned residue (22 g) was chromatographed on a silica gel open column eluting with a gradient of chloroform–MeOH (10:1 to 1:1) to give 4 fractions (Fr.1–Fr.4). Fr.2 (12.22 g) was further separated by RP-SP-HPLC eluting with MeCN–H_2_O (35:65) to afford 3 subfractions (Fr.2.1–Fr.2.3). Fr.2.1 (4.76 g) was purified by RP-SP-HPLC eluting with MeCN–H_2_O (35:65) to yield prototype 20(*S*)-G-Rg_2_ (698 mg, retention time (*t*_R_) = 27 min) and **M2** (23 mg, *t*_R_ = 29 min), respectively. Fr.3 (5.32 g) was further purified by RP-SP-HPLC eluting with MeCN–H_2_O (35:65) to give **M1** (8 mg, *t*_R_ = 8 min) and **M3** (12 mg, *t*_R_ = 19 min), respectively.

Ginsenotransmetin A (**M1**; 6-*O*-α-l-rhamnopyranosyl-(1→2)-β-d-glucopyranosyl-dammar-24*Z*- ene-3β,6α,12β,20(*R*),27-pentol): White amorphous powder; ${\left[\alpha \right]}_{D}^{20}$ +12.8 (*c* 0.185, MeOH); IR (KBr) *ν*_max_: 3417, 2935, 1643, 1462, 1384, 1129, 1075, 1048, 812 cm^−1^; ^1^H-NMR (400 MHz, py-*d*_5_) and ^13^C-NMR (100 MHz, py-*d*_5_) data see Table 1; ESI-TOF-MS *m/z* 799.56 [M − H]^−^, 845.60 [M + HCOOH − H]^−^; HR-ESI-MS *m/z* 845.4896 [M + HCOOH − H]^−^ (calculated for C_43_H_73_O_16_, 845.4899).

### 3.5. SIRT1-NAD/NADH Enzyme-Based Assay

The SIRT1 fluorometric drug discovery kit was purchased from Enzo Life Sciences Inc. (Farmingdale, NY, USA). The assay was conducted carried out according to the protocols provided by the kit instruction. The SIRT1 enzyme reaction was conducted with 50 μL per well in a half-volume 96 well microplate (Corning Costar^®^ #400012; Cambridge, MA, USA). The SIRT1 reaction solution included 0.02 U/μL enzyme, with or without test compound, in SIRT1 assay buffer. The enzyme-compound mixture and the assay buffer (25 μL) were incubated at room temperature for 5 min. The reaction was started with the addition of 25 μL of a solution containing 500 μM NAD^+^ and 100 μM Fluor de Lys-SIRT1. After 45 min of incubation at room temperature, the reaction was quenched by adding 50 μL of Fluor de Lys Developer II with 2 mM nicotinamide in the HDAC assay buffer. After 30 min of incubation, the fluorescence of the reaction solution was measured using a SpectraMax GEMNI XPS microplate reader (Molecular Devices, Sunnyvale, CA, USA.) using an excitation wavelength of 360 nm and an emission of 460 nm, after adding 50 μL of the SIRT1 assay buffer. The positive and negative controls were the reaction with dimethyl sulfoxide (DMSO) or without enzyme, respectively.

### 3.6. Chromatographic and Mass Spectrometric Conditions

Chromatographic analysis was carried out by an Agilent 1290 UPLC system (Agilent Technologies, Waldbronn, Germany), equipped with a binary pump, an online vacuum degasser, an autosampler and a thermostatically controlled column compartment. Chromatographic separation was performed by an Agilent ZORBAX RRHD Eclipse Plus C_18_ column (100 mm × 3 mm, 1.8 µm) connected to a Phenomenex Security Guard^™^ ULTRA Cartridge. The mobile phase consisted of H_2_O (A) and MeCN (B), using a gradient elution of 20%–30% B at 0–2 min; 30%–90% B at 3–18 min. The flow rate was 0.8 mL/min and the split ratio was 1:1. The detection wavelength was set at 203 nm and the temperature was set at 45 °C. The inject volume was 1 µL.

Detection was carried out by an Agilent Q-TOF 6540, equipped with Agilent Jet Stream (AJS) ESI source and data was collected using Agilent MassHunter Workstation Data acquisition software. The instrument was operated in both positive and negative ion modes. The MS parameters were set as follows: in negative mode, gas temperature: 300 °C, gas flow: 5 L/min, nebulizer pressure: 35 psi, sheath gas temperature: 400 °C, sheath gas flow: 12 L/min, capillary voltage: 3500 V, nozzle voltage: 1500 V, fragmentor voltage: 280 V, two collision-energy voltage: 40 V and 60 V. Internal references (Purine and HP-0921) were adopted to modify the measured masses in real time, and the reference masses in negative ion mode was at *m/z* 119.0363 and 1033.9881. The mass spectrometer was in full scan range of *m/z* 100–1700 for MS and MS/MS.

### 3.7. Statistical Analysis

All experimental results were presented as mean ± SD. The Student’s *t*-test was used for comparisons among various treatment groups and compounds. The level of statistical significance was taken when *p* < 0.05.

## 4. Conclusions

In this study, the metabolic profiles of 20(*S*)-G-Rg_2_ (**1**) in a RLM incubation system were qualitatively described for the first time. The metabolites were isolated by CC and identified by spectroscopic and UPLC-Q-TOF/MS methods. 20(*S*)-G-Rg_2_ could be converted into a new metabolite, ginsenotransmetin A, and mono- and dioxidations as well as cyclization metabolites, and so on. Partial metabolic pathways were proposed. The greater significance of the present study is that it clarified for the first time the potential bioactive form of **1**, as well as the fact that **1** and its some metabolites are potential SIRT1 activators. With the identification of metabolites and their metabolic mechanism, the application value of **1** would be significantly elevated. The results provided a meaningful basis for the clinical application of **1** and a comprehensive understanding of the metabolic fate of **1** in the liver. The metabolites **M3** and **M4** could serve as potential lead molecules to develop new drugs for the treatment of aging diseases associated with the activation of SIRT1 [52].

## Figures and Tables

**Table 1:** ^1^H- and ^13^C-NMR data in pyridine-*d*_5_ (δ_ppm_) of aglycone parts of **M1** and **M2** ^a^.

No.	M1 (Ginsenotransmetin A)		M2 (20(*R*)-Ginsenoside Rg_2_)	
	^1^H (*J* in Hz)	^13^C	^1^H (*J* in Hz)	^13^C
1α	0.95 (1H, m)	39.8t	0.95 (1H, m)	39.8t
1β	1.60 (1H, m)		1.60 (1H, m)	
2α	1.85 (1H, m)	27.9t	1.85 (1H, m)	27.9t
2β	1.77 (1H, m)		1.77 (1H, m)	
3β	3.42 (1H, dd, 11.7, 4.1)	78.7d	3.44 (1H, dd, 11.5, 4.1)	78.8d
4	–	40.2s	–	40.2s
5α	1.43 (1H, d, 10.7)	61.0d	1.44 (1H, d, 10.7)	61.0d
6β	4.64 (1H, br dd, 10.7, 3.3)	74.3d	4.65 (1H, br dd, 10.7, 3.1)	74.5d
7α	1.96 (1H, t, 10.7)	46.2t	1.97 (1H, t, 10.7)	46.2t
7β	2.29 (1H, dd, 10.7, 3.1)		2.30 (1H, dd, 10.7, 3.1)	
8	–	41.3s	–	41.3s
9α	1.48 (1H, br d, 10.9)	49.9d	1.48 (1H, br d, 11.3)	50.7d
10	–	39.5s	–	39.5s
11α	2.09 (1H, m)	32.4t	2.12 (1H, m)	32.4t
11β	1.50 (1H, m)		1.54 (1H, m)	
12α	3.93 (1H, m)	71.2d	3.93 (1H, m)	71.1d
13β	1.92 (1H, t, 10.1)	48.3d	2.01 (1H, t, 10.3)	49.0d
14	–	51.8s	–	51.9s
15α	1.46 (1H, m)	31.4t	1.56 (1H, m)	31.6t
15β	0.86 (1H, m)		0.93 (1H, m)	
16α	1.40 (1H, m)	26.9t	1.83 (1H, m)	26.8t
16β	1.21 (1H, m)		1.56 (1H, m)	
17α	2.32 (1H, m)	50.7d	2.33 (1H, m)	49.9d
18β	1.19 (3H, s)	17.3q	1.19 (3H, s)	17.4q
19β	0.93 (3H, s)	17.8q	0.94 (3H, s)	17.8q
20	–	73.1s	–	73.2s
21α	1.29 (3H, s)	22.0q	1.31 (3H, s)	22.9q
22a	1.74 (1H, m)	42.9t	1.78 (1H, m)	43.4t
22b	1.60 (1H, m)		1.68 (1H, m)	
23a	2.65 (1H, m)	22.8t	2.54 (1H, m)	22.8t
23b	2.39 (1H, m)		2.46 (1H, m)	
24	5.43 (1H, t, 7.5)	128.2d	5.24 (1H, t, 6.8)	126.2d
25	–	136.2s	–	130.9s
26	1.96 (3H, s)	22.0q	1.63 (3H, s)	26.0q
27	4.44 (1H, br d, 12.4)	61.0t	1.58 (3H, s)	17.8q
	4.51 (1H, br d, 12.4)			
28β	2.07 (3H, s)	32.4q	2.07 (3H, s)	32.4q
29α	1.30 (3H, s)	17.8q	1.32 (3H, s)	17.9q
30α	0.91 (3H, s)	17.1q	0.93 (3H, s)	17.4q

^a^ ^1^H-NMR chemical shift values (δ_ppm_) followed by multiplicity. Assignments were based on ^1^H–^1^H COSY, NOESY, HSQC, and HMBC experiments. s: C; d: CH; t: CH_2_, q: CH_3_.

**Table 2:** ^1^H- and ^13^C-NMR data in pyridine-*d*_5_ (δ_ppm_) of glycosyl parts of **M1** and **M2** ^a^.

No.	M1 (Ginsenotransmetin A)		M2 (20(*R*)-Ginsenoside Rg_2_)	
	^1^H (*J* in Hz)	^13^C	^1^H (*J* in Hz)	^13^C
6-Glc				
1′	5.21 (1H, d, 6.4)	102.1d	5.23 (1H, d, 6.8)	102.1d
2′	4.34 (1H, dd, 9.1, 6.4)	79.6d	4.38 (1H, dd, 9.0, 6.8)	79.6d
3′	4.32 (1H, dd, 9.1, 8.3)	78.5d	4.36 (1H, dd, 9.0, 8.4)	78.6d
4′	4.16 (1H, dd, 9.0, 8.2)	72.8d	4.21 (1H, dd, 9.2, 8.4)	72.8d
5′	3.92 (1H, br dd, 8.2, 5.6)	78.6d	3.96 (1H, br dd, 8.4, 5.6)	78.5d
6′a	4.35 (1H, dd, 11.5, 5.6)	63.3t	4.38 (1H, dd, 11.5, 5.6)	63.3t
6′b	4.48 (1H, dd, 11.5, 2.3)		4.54 (1H, dd, 11.5, 2.3)	
2-′Rha				
1′′	6.44 (1H, brs)	101.9d	6.45 (1H, brs)	101.9d
2′′	4.74 (1H, br d, 3.9)	72.4d	4.80 (1H, br d, 3.7)	72.3d
3′′	4.63 (1H, dd, 9.6, 3.9)	72.6d	4.67 (1H, dd, 9.5, 3.7)	72.6d
4′′	4.31 (1H, dd, 9.6, 2.2)	74.4d	4.33 (1H, dd, 9.5, 2.1)	74.4d
5′′	4.93 (1H, dd, 9.5, 6.2)	69.6d	4.96 (1H, dd, 9.5, 6.1)	69.6d
6′′	1.74 (1H, d, 6.2)	18.9q	1.75 (1H, d, 6.2)	18.9q

^a^ ^1^H-NMR chemical shift values (δ_ppm_) followed by multiplicity. Assignments were based on ^1^H–^1^H COSY, NOESY, HSQC, and HMBC experiments. s: C; d: CH; t: CH_2_, q: CH_3_.

## Supplementary Materials

The typical mass spectra and possible fragmentations of 20(*S*)-G-Rg_2_ and pseudoginsenoside F_11_, and ^1^H- and ^13^C-NMR data of 20(*S*)-G-Rg_2_ and **M3** are available as Supplementary Materials, which may be accessed at: http://www.mdpi.com/1420-3049/21/6/757/s1.
