# Supplementary material for: Metabolism of 20(S)-Ginsenoside Rg2 by Rat Liver Microsomes: Bioactivation to SIRT1-Activating Metabolites
Source: Molecules. 2016 Jun 10;21(6):757. doi: 10.3390/molecules21060757 (PMC6273440; doi:10.3390/molecules21060757)
Supplement: Supplementary file 1 [file molecules-21-00757-s001.pdf]

Supplementary Materials: Metabolism of 20(*S*)-Ginsenoside Rg2 by Rat Liver Microsomes: Bioactivation to Metabolites of Activating SIRT1

Li-Yuan Ma, Qi-Le Zhou, Xin-Bao Yang, Hong-Ping Wang and Xiu-Wei Yang

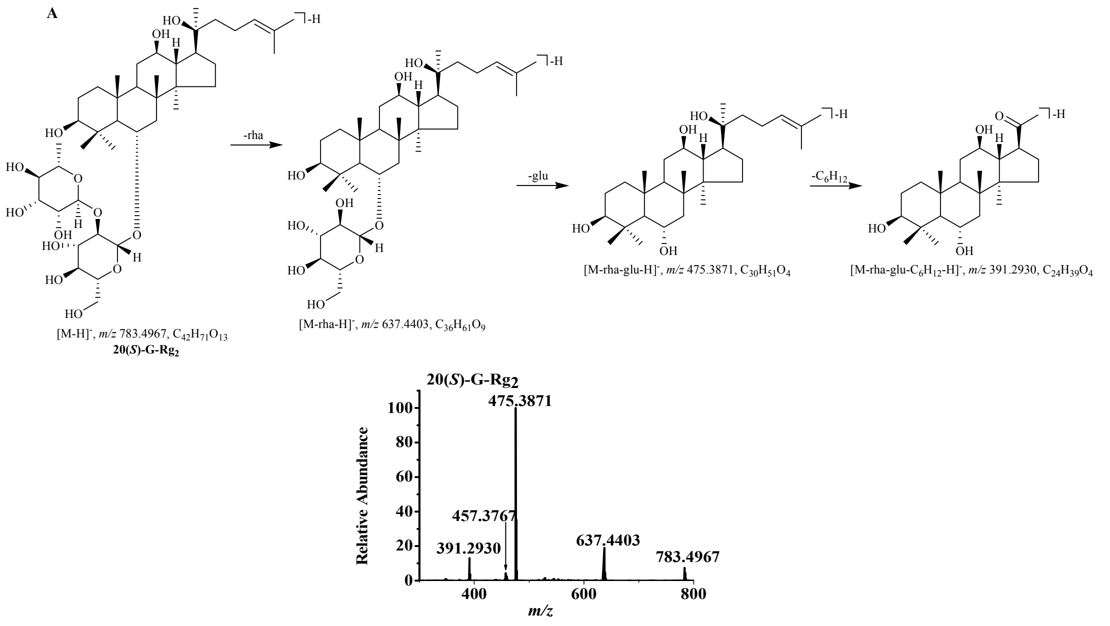

(**A**)

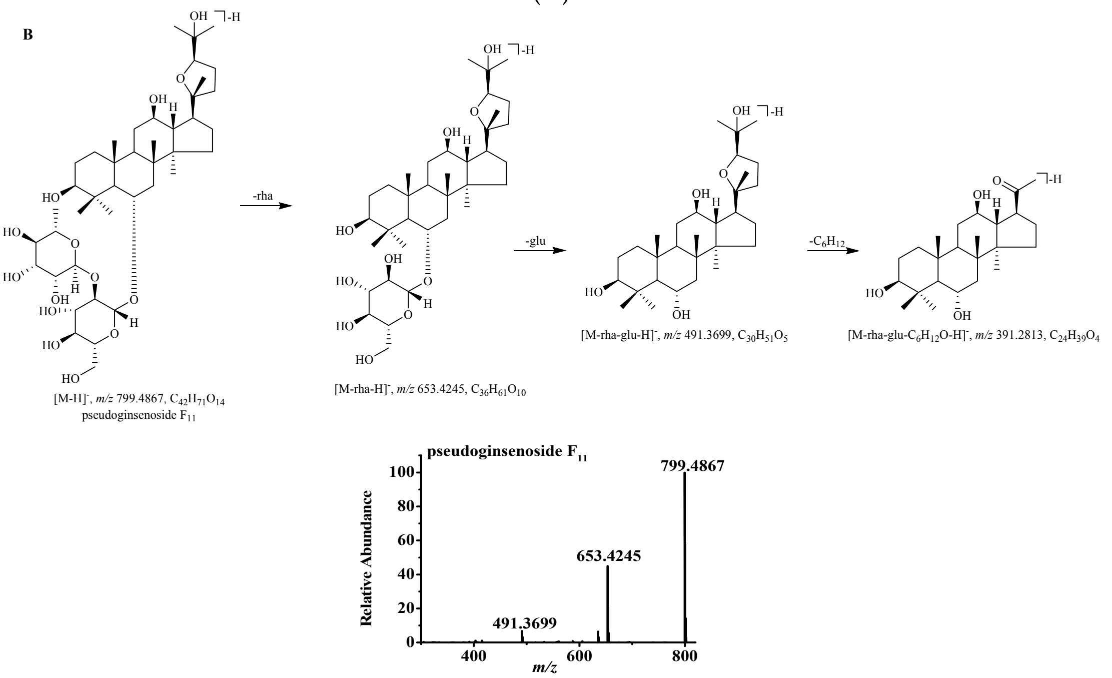

(**B**)

Figure S1. The typical mass spectra and possible fragmentations of 20(*S*)-G-Rg2 (**A**) and pseudoginsenoside F11 (**B**).

Table S1. ^{1}H (400 MHz) and ^{13}C (100 MHz) NMR data in pyridine-d_5 (δ_{ppm}) of **1** and **M3** [a].

No.	20(*S*)-Ginsenoside-Rg2 (1)		Pseudoginsenoside F11 (M3)	
	^{1}H (*J* in Hz)	^{13}C	^{1}H (*J* in Hz)	^{13}C
1α	0.96 (1H, m)	39.7t	0.96 (1H, m)	39.7t
1β	1.60 (1H, m)		1.62 (1H, m)	
2α	1.85 (1H, m)	28.0t	1.86 (1H, m)	27.8t
2β	1.77 (1H, m)		1.77 (1H, m)	
3β	3.46 (1H, dd, 11.1, 4.3)	78.9d	3.48 (1H, dd, 11.4, 4.8)	78.5d
4	–	40.2s	–	40.2s
5α	1.40 (1H, d, 11.1)	61.1d	1.41 (1H, d, 10.7)	61.0d
6β	4.66 (1H, br dd, 11.1, 3.0)	74.7d	4.72 (1H, br dd, 10.7, 3.1)	74.4d
7α	1.98 (1H, t, 10.6)	46.3t	1.93 (1H, t, 12.6)	46.1t
7β	2.27 (1H, dd, 10.6, 3.0)		2.27 (1H, dd, 12.6, 3.1)	
8	–	39.9s	–	41.2s
9α	1.48 (1H, br d, 12.0)	50.0d	1.48 (1H, dd, 12.6, 2.4)	50.2d
10	–	41.4s	–	39.6s
11α	2.12 (1H, m)	32.3t	2.06 (1H, m)	32.6t
11β	1.81 (1H, m)		1.27 (1H, m)	
12α	3.93 (1H, m)	71.3d	3.71 (1H, td, 9.9, 4.3)	71.3d
13	2.00 (1H, t, 10.3)	48.5d	2.17 (1H, t, 9.9)	48.4d
14	–	51.9s	–	52.3s
15α	1.54 (1H, m)	31.6t	1.42 (1H, m)	32.9t
15β	1.45 (1H, m)		0.89 (1H, m)	
16α	1.84 (1H, m)	27.1t	2.14 (1H, m)	25.6t
16β	1.55 (1H, m)		1.87 (1H, m)	
17α	2.30 (1H, m)	54.9d	1.78 (1H, m)	49.6d
18β	1.38 (3H, s)	17.2q	1.21 (3H, s)	17.0q
19β	0.95 (3H, s)	18.0q	0.95 (3H, s)	18.0q
20	–	73.3s	–	86.8s
21α	1.38 (3H, s)	27.3q	1.25 (3H, s)	27.1q
22a	2.01 (1H, m)	36.1t	1.79 (1H, dd, 12.5, 3.7)	31.8t
22b	1.64 (1H, m)		1.58 (1H, dt, 12.5, 3.9)	
23a	2.57 (1H, m)	23.2t	1.85 (1H, m)	28.9t
23b	2.27 (1H, m)		1.30 (1H, dt, 10.1, 7.9)	
24	5.32 (1H, t, 6.6)	126.6d	3.94 (1H, t, 7.8)	85.8d
25	–	131.0s	–	70.5s
26	1.67 (3H, s)	26.1q	1.26 (3H, s)	27.1q
27	1.62 (3H, s)	17.9q	1.46 (3H, s)	27.3q
28β	2.07 (3H, s)	32.4q	2.11 (3H, s)	32.3q
29α	1.33 (3H, s)	17.9q	1.34 (3H, s)	17.7q
30α	0.90 (3H, s)	17.4q	0.91 (3H, s)	18.3q
		6-Glc		
1′	5.22 (1H, d, 6.8)	102.14d	5.26 (1H, d, 6.9)	102.1d
2′	4.35 (1H, dd, 8.9, 6.8)	79.6d	4.38 (1H, dd, 9.0, 6.9)	79.6d
3′	4.33 (1H, dd, 8.9, 8.4)	78.7d	4.36 (1H, dd, 9.0, 8.4)	78.7d
4′	4.18 (1H, dd, 8.9, 8.4)	72.9d	4.21 (1H, dd, 9.2, 8.4)	72.8d
5′	3.93 (1H, br dd, 8.4, 5.0)	78.6d	3.96 (1H, br dd, 8.4, 5.6)	78.6d
6′a	4.34 (1H, dd, 11.2, 5.3)	63.4t	4.38 (1H, dd, 11.5, 5.6)	63.3t
6′b	4.49 (1H, dd, 11.2, 2.3)		4.54 (1H, dd, 11.5, 2.3)	
		Rha		
1″	6.43 (1H, brs)	102.05d	6.49 (1H, brs)	101.9d
2″	4.76 (1H, br d, 3.6)	72.5d	4.80 (1H, br d, 3.7)	72.4d
3″	4.66 (1H, dd, 9.8, 3.6)	72.6d	4.67 (1H, dd, 9.5, 3.7)	72.6d
4″	4.32 (1H, dd, 9.8, 2.1)	74.4d	4.33 (1H, dd, 9.5, 2.1)	74.3d
5″	4.96 (1H, dd, 9.8, 5.6)	69.7d	4.96 (1H, dd, 9.5, 6.1)	69.6d
6″	1.75 (1H, d, 5.6)	19.0q	1.79 (1H, d, 6.2)	18.9t
